# The Multifaceted Nature of Nucleobindin-2 in Carcinogenesis

**DOI:** 10.3390/ijms22115687

**Published:** 2021-05-26

**Authors:** Anna Skorupska, Rafał Lenda, Andrzej Ożyhar, Dominika Bystranowska

**Affiliations:** Department of Biochemistry, Molecular Biology and Biotechnology, Faculty of Chemistry, Wrocław University of Science and Technology, Wybrzeże Wyspiańskiego 27, 50-370 Wrocław, Poland; anna.skorupska@pwr.edu.pl (A.S.); rafal.lenda@pwr.edu.pl (R.L.); andrzej.ozyhar@pwr.edu.pl (A.O.)

**Keywords:** Nucb2, nesfatin-1, carcinogenesis, apoptosis, biomarker, tumors

## Abstract

Nucb2 is a multifunctional protein associated with a variety of biological processes. Multiple studies have revealed that Nucb2, and its derivative nesfatin-1, are involved in carcinogenesis. Interestingly, the role of Nucb2/nesfatin-1 in tumorigenesis seems to be dual—both pro-metastatic and anti-metastatic. The implication of Nucb2/nesfatin-1 in carcinogenesis seems to be tissue dependent. Herein, we review the role of Nucb2/nesfatin-1 in both carcinogenesis and the apoptosis process, and we also highlight the multifaceted nature of Nucb2/nesfatin-1.

## 1. Introduction

Nucleobindin-2 (Nucb2) is a DNA/Ca^2+^-binding protein, the characteristic structure of which consists of several functional domains: a signal peptide, a Leu/Ile-rich region, a DNA-binding domain, two EF-hand domains, an acidic-rich region, and a leucine zipper motif [1]. Oh-I et al. [2] showed that Nucb2 can be proteolytically converted into three peptide segments: nesfatin-1, -2, and -3. Additionally, nesfatin-1 turned out to be involved in food intake inhibition [2], whereas the function of the two other parts of Nucb2 still remains an open question. The high expression levels of Nucb2 and/or nesfatin-1 (Nucb2/nesfatin-1) were originally found in the hypothalamus [2]. However, Nucb2/nesfatin-1 is also widely expressed in a variety of peripheral tissues—e.g., adipose tissue [3], the pancreas [4], and the reproductive system [5]—which suggests the implication of Nucb2/nesfatin-1 in controlling energy homeostasis processes. Interestingly, Nucb2/nesfatin-1 has an insulin-dependent anti-hyperglycemic effect in mice [6]. Nucb2 was also found to be involved in multiple interactions, e.g., with the tumor necrosis factor receptor 1 [7] and the growth suppressor necdin protein [8]. Recently, Nucb2s from *Homo sapiens* and *Gallus gallus* were characterized as proteins that exhibit the properties of inherently disordered proteins (IDPs) [9]. The altered expression of IDPs is involved in the pathogenesis of several diseases, such as Alzheimer’s disease [10] and cancer [11]. In addition to its physiological role, Nucb2/nesfatin-1 is also associated with pathological states, such as carcinoma development [12,13]. In recent years, an increasing number of research studies have focused on this relationship [13,14,15]. The purpose of this review is to discuss the multifaceted nature of Nucb2/nesfatin-1 in tumorigenesis. 

## 2. Nucb2 Involvement in Cancer Progression

According to the World Health Organization (WHO), cancer is the second leading cause of death, and was responsible for 9.6 million deaths globally in 2018. The major reason for mortality (about 90%) is cancer metastasis, and not the primary tumor itself [16]. Cancer is usually considered to be a dynamic system, and malignant tumors, with their ability to invade and metastasize, also develop. This results in the formation of tumors that are heterogeneous in nature [17], which is what makes cancers so dangerous. Nowadays, biomarkers can be used at each stage of cancer progression and for monitoring the efficacy of therapy [18]. Therefore, the necessity of searching for cancer biomarkers is urgent.

Proteins with multiple domains have been shown to be essential to numerous signaling pathways. A number of studies show that Nucb2 might be associated with diverse cellular functions. Despite the fact that its broad physiological function has been extensively investigated, there is also growing evidence that Nucb2 may play an important role in the multistep processes of tumorigenesis. A high expression of Nucb2, in comparison to adjacent non-cancerous cells, was found in breast [13], prostate [12], colon [19], endometrial [14], papillary thyroid [20], and renal cell carcinomas [21]. The immunoreactivity of Nucb2 was mainly detected in the cytoplasm of cancer cells [13,22,23]. However, in glioblastoma cancer cells [24], the localization of Nucb2 was nuclear. In most cases, Nucb2 expression was correlated with the key clinicopathological characteristics of cancer. In clear-cell renal cell carcinoma (ccRCC), the high level of Nucb2 expression was linked to a progressed tumor stage and metastasis [22]. A similar pattern was observed for prostate [12] and colon [25] cancer cells. The high level of Nucb2 mRNA expression was related to a higher Gleason score, a higher level of preoperative prostate-specific antigen (PSA), positive lymph node metastasis, and angiolymphatic invasion [12]. Additionally, patients with colon carcinomas with TNM Classification of Malignant Tumors of stages III-IV have an increased expression of Nucb2 when compared with patients with stages I-II [25]. Interestingly, Nucb2 overexpression was also associated with a poor prognosis. Patients with strong Nucb2 tumor expression had a shorter overall survival rate [22,26] and increased incidence of recurrence [13]. The prostate cancer patients with Nucb2 overexpression had a shorter biochemical recurrence-free period [23]. These data indicate that a high level of Nucb2 expression may become a new prognostic factor in cancer. The analysis of the expression level of Nucb2 might be utilized in cancer therapy monitoring, as well as provide independent information alongside known biomarkers, such as PSA. 

Upregulation of Nucb2 expression was also significantly associated with lymph node metastasis in breast carcinoma cells [13]. Breast cancer progression is associated with the steroid hormone estrogen [27]. Estrogen mediates its effects by binding to estrogen receptors (ERs) [28]. The expression of ERs was found in 75% of all breast cancer cases [29,30]. ERs, in response to estrogens, activate the expression of the autocrine, paracrine, and intracrine protein growth factors by binding to estrogen response elements (EREs) located in the promoted region of their genes [31]. Interestingly, analysis of microarray experiments of gene expression profiling showed that the *Nucb2* gene is among the genes involved in the recurrence in estrogen receptor (ER)-positive breast carcinoma patients after surgery [13]. Additionally, high-affinity EREs were identified in the promoter region (at a range a of –10 kb to +5 kb from mRNA 5’-ends) of human *Nucb2* [32]. Further research showed that estradiol treatment of the MCF-7 breast cancer cell line for 3 days significantly increased the Nucb2 expression level [13]. However, the estradiol and ICI 182780 (a potent antagonist of ERs) treatment lessened the Nucb2 mRNA expression to a level lower than the basal level. The expression of Nucb2 in MCF-7 cells is probably ER-regulated [13]. All in all, the above discussed results suggest that *Nucb2* is considered to be an oncogene in ER-induced breast carcinoma [13]. 

Further studies also revealed that Nucb2 is involved in cancer progression and metastasis. Bladder cancer cell lines [26], transfected with Nucb2-targeted shRNA to knockdown Nucb2 expression, were characterized in order to verify the effects of Nucb2 on cell proliferation and invasion. The results of the assays revealed that the knockdown of Nucb2 with special shRNA inhibits invasion and proliferation in bladder cancer cells. Downregulation of Nucb2 expression also induces the decreased expression of two other proliferation markers: Ki67, and the proliferating cell nuclear antigen [33]. The results of an additional wound-healing assay revealed that Nucb2 also had a stimulatory effect on cell migration [26]. Additionally, the same research group analyzed whether there was a correlation between Nucb2-regulated cell migration and the significance of Nucb2 in the migration of cancer cells facilitated by the action of matrix metalloproteases MMP-2 and MMP-9. Overexpression of MMP-2 [34] and MMP-9 [34,35] is highly correlated with tumor dissemination and invasiveness. The expression levels of MMP-2 and MMP-9 in bladder cancer cells transfected with Nucb2 shRNA was lowered, indicating that Nucb2 may affect cancer migration and invasion through the MMP-2 and MMP-9 signaling pathways [26]. Analogous correlation was also recently found in papillary thyroid tumor cells [20], as well as previously for breast [13], renal [21], and colon [19] cancer cells. Similarly, in endometrial carcinoma cells [14], Nucb2 and its derivative nesfatin-1 significantly induced cell proliferation and migration. Moreover, in vivo studies also showed that the knockdown of Nucb2 might decrease tumor growth, at least in the case of thyroid and bladder cancer cells [20,26]. In addition, lung metastases were not observed in cells with Nucb2 suppression [24,26]. All of the above-mentioned analyses indicate that Nucb2 is linked in a tissue-specific manner to tumor development and metastasis, both in vitro and in vivo.

### 2.1. The Action of Transcription Factors on the Migration and Invasion of Nucb2-Mediated Cancer Cells

**Figure 1:**
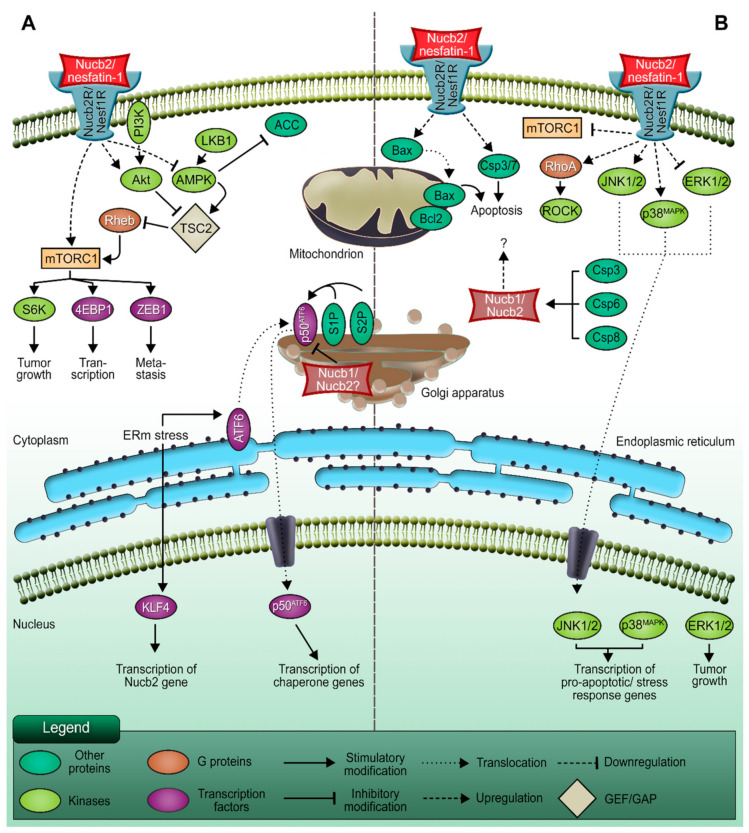
The dual role of Nucb2/nesfatin-1 in carcinogenesis. (**A**) The pro-metastatic function of Nucb2/nesfatin-1. (**B**) The involvement of Nucb2/nesfatin-1 in apoptosis processes. Nucb2 mediates metastasis through the LKB1/AMPK/mTORC1/ZEB1 pathways. Nucb2 is thought to participate in the adaptation of cancer cells to ERm stress, which also promotes cell metastasis. On the other hand, Nucb2/nesfatin-1 is implicated in the apoptosis process through such pathways as the mTOR/RhoA/ROCK and JNK-1/2/p38^MAPK^ pathways. Apoptosis induction by Nucb2/nesfatin-1 is also exerted by the regulation of apoptotic gene expression, e.g., Bax and Bcl-2. Additionally, Nucb2 is a substrate of caspase cleavage, which also indicates the participation of Nucb2 in apoptosis.

Epithelial–mesenchymal transition (EMT) is a cellular program that converts the apical–basal polarity of epithelial cells with a cell–cell junction to mesenchymal cells with higher migrating and invasive properties [36]. The accelerated motility of these mesenchymal cells indicates that EMT may in turn contribute to the metastasis process [37]. The exact mechanism of the pathogenesis of Nucb2 has not yet been explained in detail; there are, however, some clues. As the knockdown of *Nucb2* in the lymph node metastases of SW620 colon cancer cells leads to the modulation of their shape, this might suggest the involvement of Nucb2 in the EMT [19]. Moreover, further studies based on the microarray assays of various human tumor samples demonstrated that the suppression of Nucb2 expression in the SW620 cells resulted in a low level of zinc finger E-box-binding homeobox transcription factor 1 (ZEB1), twist family bHLH transcription factor 1 (Twist), and snail family zinc finger 2 (Slug)—three transcription factors engaged in the EMT [19]. The upregulation of markers characteristic of epithelial cells—such as E-cadherin, β-catenin, and claudin-3—has also been observed in Nucb2-knockdowned SW620 cells. Additionally, ZEB1 overexpression counteracted the migration ability inhibition caused by the knockdown of Nucb2 expression in SW620 cells, and also led to the enhanced expression of the metastatic promotor N-cadherin and a decreased level of the migration suppressor E-cadherin [19]. Thus, Nucb2 mediates the EMT in colon cancer cells through the ZEB1, Twist, and Slug pathways (Figure 1A) [19]. 

**Figure 2:**
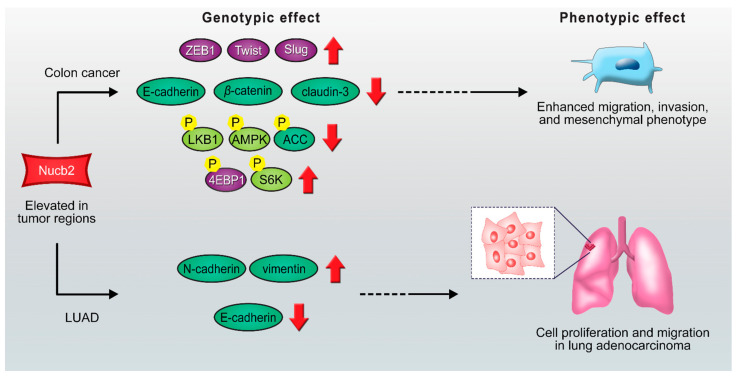
Scheme of proposed Nucb2 mediated signaling pathways in colon cancer and LUAD. Nucb2 enhances migration, invasion, and mesenchymal phenotype in colon cancer through the LKB1/AMPK/TORC1/ZEB1 pathways. Nucb2 also plays an oncogenic role in LUAD by increasing the expression of N-cadherin and vimentin and promoting the deficiency of the E-cadherin level. See the text for more details.

Generally, deregulation of the activity of signaling pathways is implicated in cancer progression. The studies presented by Kan et al. [19] revealed the association of Nucb2-induced colon cancer metastasis with the liver kinase B1 (LKB1), 5’AMP-activated protein kinase (AMPK), and mTOR (mammalian target of rapamycin) pathways [19]. It has been found that enhanced phosphorylation of LKB1, AMPK, and acetyl-CoA carboxylase, and diminished phosphorylation of S6 kinase and eukaryotic translation initiation factor 4E (eIF4E)-binding protein 1, are caused by Nucb2 depletion in the SW620 cells [19]. Interestingly, the phosphorylation pattern was inverted upon the addition of the AMPK inhibitor. Moreover, utilization of the AMPK inhibitor results in the upregulation of ZEB1 expression, the reduction of the E-cadherin level, and the escalation of the migration and invasiveness of SW620 cells with the suppression of Nucb2 expression [19]. These results indicate that *Nucb2* might play an oncogene role in colon cancer cells (Figure 2). Nucb2 promotes migration, invasion, and EMT through the LKB1/AMPK/mTORC1/ZEB1 pathways (see Figure 1). Nucb2 inhibits the LKB1/AMPK route with simultaneous enhancement of the mTOR pathway. The mTOR pathway probably participates in the activation of ZEB1 [19]. Interestingly, the association of EMT promotion by Nucb2 with the AMPK/mTORC1/ZEB1 pathways has also been reported for renal cell carcinoma cells [38]. In addition, in endometrial cancer cells, nesfatin-1 activates the mTOR pathway through phosphorylation [14]. 

Furthermore, a high level of Nucb2 expression was found in lung adenocarcinoma (LUAD), which is the predominant type of non-small-cell lung carcinoma [39]. *Nucb2* has been presented as the oncogene implicated in the LUAD progression pathways [39]. The deciphered mechanism was based on the upregulation of FTX, which is a long non-coding RNA [39]. FTX inhibited the activity of microRNA-335-5p, while the depletion of microRNA-335-5p resulted in increased expression of Nucb2 [39]. Both Nucb2 and FTX induced the processes implicated in LUAD progression. FTX and Nucb2 increased the expression of N-cadherin and vimentin and promoted the deficiency of the E-cadherin level, which also indicates their function in inducing the EMT process (Figure 2). It was also shown that FTX/Nucb2 accelerated the metastasis of cancer through mediation in the phosphorylation of protein kinase B and mTOR [39]. Remarkably, the involvement of mTOR in the regulation of Nucb2 expression has previously been reported in the stomach [40,41]. 

Hence, Nucb2 participates in colon cancer cell and LUAD metastasis through EMT induction. Interestingly, in both presented cases, Nucb2 impacts cancer progression via the mTOR pathway. 

### 2.2. The Potential Role of Nucb2 in Melanoma Metastasis under Endoplasmic Reticulum (ERm) Stress

Recently, Nucb2 was also reported to involve metastasis in the melanoma cells by adapting to endoplasmic reticulum (ERm) stress [42]. One study showed that ERm stress induces the expression of Krüppel-like factor 4 (KLF4) [42], which is a zinc-finger-type transcription factor with an oncogenic role. KLF4 was further found to bind to the promoter of Nucb2, facilitating its transcription, and through the regulation of the expression of Nucb2 in vivo and in vitro it was shown to be involved in the promotion of cell metastasis and the inhibition of apoptosis processes under ERm stress [42]. The increased expression of KLF4 was therefore associated with a high level of Nucb2 [42]. Although an elevated level of Nucb2 was shown to be linked to the inhibition of ERm-stress-induced apoptosis and the promotion of cell metastasis in melanoma, the detailed regulations governing the downstream pathway of Nucb2 still need to be investigated. Interestingly, a possible link between nucleobindins and ERm stress responses has previously been suggested for Nucb1, a paralog of Nucb2 [43]. Findings that the *Nucb1* gene was identified as being ERm stress inducible were reported in research on the activating transcription factor 6 (ATF6), the activation of which during ERm stress turned out to be suppressed by overexpression of Nucb1. As illustrated in Figure 1, an ERm-membrane-anchored ATF6 is transported to the Golgi apparatus and cleaved by site-1 protease (S1P) in order to activate the unfolded protein response (UPR). The researchers identified Nucb1 as a repressor of this S1P-mediated ATF6 activation, and showed that the knockdown of Nucb1 by siRNA accelerates ATF6 cleavage during ERm stress. Moreover, it was shown that the *Nucb1* promoter region possesses two ATF6 response elements, which might be utilized for transcriptional activation of ATF6 or other factors [43]. The function of ATF6 in cancer progression seems contradictory. Various studies have shown that ATF6 is implicated in cancer cell survival [44,45,46]. However, it was also presented that ATF6 mediates the apoptosis processes in myoblast cells [47]. The 62% sequence identity of amino acids Nucb1 and Nucb2 [48], and also the Golgi location of both paralogs [49,50,51], may suggest—but does not need to—that Nucb1 and Nucb2 have at least partially similar roles. 

The findings presented above indicate that Nucb2 might be involved in melanoma cell metastasis through ERm stress induction. However, the downstream pathways of these actions require further research. 

### 2.3. Nucb2 Expression during Cancer-Associated Anorexia-Cachexia

Cancer anorexia–cachexia syndrome (CACS) is a disorder characterized by decreased food nutritional intake, tissue wasting, anorexia, and the loss of muscle, adipose tissue, and body mass [52]. This state occurs in 15–40% of cancer patients, and in 80% of advanced cancer stage patients, in turn impacting the quality of their lives, decreasing their chances of survival, and frequently causing death [53]. The origin of CACS is multifactorial, involving mediators like cytokines (interleukin 1, interleukin 6, and tumor necrosis factor α), neuropeptides (leptin, neuropeptide Y (NPY), and ghrelin), and neurotransmitters [54,55,56]. Nesfatin-1 was described as an anorexigenic peptide [2]. Intracerebroventricular injection of nesfatin-1 results in food intake inhibition in rodents [2]. Interestingly, the implication of Nucb2/nesfatin-1 in CACS was shown by Burgos et al. [57]; their studies were conducted on a tumor-bearing murine model with implanted methylcholanthrene-induced sarcoma (MCG101), which is a low or undifferentiated epithelial-like solid tumor with high expression of prostaglandin E_2_ [58]. This model corresponds to cancer patients with CACS [59]. The tumor-bearing group of mice had an increased level of *Nucb2* mRNA in the paraventricular nucleus (PVN) compared with the control group, whereas in the arcuate nucleus and brainstem, the expression level of *Nucb2* mRNA was similar to that of the control group. The authors suggest that the induction of Nucb2 in the PVN could play a primary role in promoting the tumor-induced anorexia response of the host [57]. Additionally, anorexia in CACS might be enhanced by other factors, e.g., anxiety, pain, or depression [55,60]. It is important to acknowledge that multiple studies have shown the involvement of Nucb2/nesfatin-1 in anxiety- [61,62] and depression-like behavior [63,64] induction, and therefore Nucb2/nesfatin-1 may play a role in tumor-induced anorexia associated with CACS through variable pathways. 

Taken together, the above results reveal that Nucb2/nesfatin-1 plays a complex role in cancer metastasis. It involves multiple signaling pathways, which makes Nucb2/nesfatin-1 a significant therapeutic target. It is worth noting that Nucb2/nesfatin-1 not only stimulates cancer progression, but also influences the life quality of patients, in turn leading to decreased treatment success. Still, the control of Nucb2/nesfatin-1 activity in cancer progression requires further investigation.

## 3. Apoptotic Potential of Nucb2

Cell apoptosis is a form of programmed cell death that naturally occurs during embryogenesis, or as a result of pathological conditions, e.g., neurodegenerative diseases, autoimmune diseases, or cancer [65]. Malignant cells are insensitive to apoptotic stimuli, which results in uncontrolled proliferation and tumor growth. Many anticancer drugs are thus designed to target and activate specific components of apoptotic machinery in order to facilitate the eradication of cancer cells. Although Nucb2/nesfatin-1 seems to play a role in the progression and invasiveness of breast [13], colon [19], prostate [23], renal [21], and endometrial cancer [14], there is also evidence that increased levels of Nucb2/nesfatin-1 trigger apoptosis in other malignancies, which as a result might render this unique peptide useful in the treatment of cancer.

### 3.1. Nucb2/nesfatin-1-Induced Apoptosis in Ovarian Cancer

**Figure 3:**
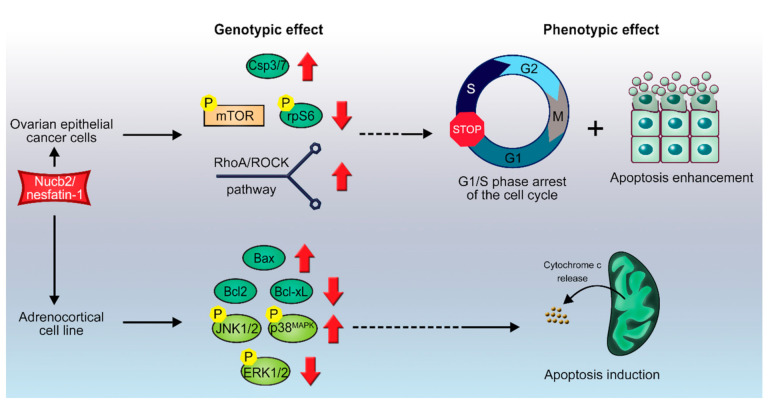
Scheme of proposed Nucb2-induced apoptosis in ovarian cancer and in adrenocortical carcinoma. Nucb2 exerts apoptosis in ovarian epithelial cell carcinoma through the mTOR/RhoA/ROCK pathways. Nucb2 also alters the phosphorylation pattern of kinases belonging to the family of serine/threonine mitogen-activated protein kinases (MAPKs), resulting in elevated levels of phosphorylated p-JNK-1/2, and p-p38^MAPK^, and decreased levels of phosphorylated p-ERK1/2. See the text for more details.

Ovarian cancer is the 5th most common cause of cancer death among women [66], and has a poor survival rate of 5 years in 93% of cases due to its late diagnosis [67]. In 2013, Xu et al. showed that Nucb2/nesfatin-1 inhibit ovarian epithelial cell carcinoma in vitro in HO-8910 cells [15]. Stimulation of HO-8910 cells for 48 h with Nucb2/nesfatin-1 results in a concentration-dependent G1/S phase arrest of the cell cycle and enhanced apoptosis, which is shown by increased levels of caspase-3/7. This effect is evoked by downregulation of the mTOR pathway (Figure 3), leading to decreased levels of the phosphorylated mTOR and S6 ribosomal proteins [15]. Unexpectedly, upregulation of the Ras homologue gene family member A/Rho-associated coiled-coil-containing kinases (RhoA/ROCK) pathway was also observed under Nucb2/nesfatin-1 treatment. Conversely, inhibition of the RhoA/ROCK pathway by the Y27632 inhibitor revoked pro-apoptotic action of Nucb-2/nesfatin-1 in HO-8910 cells [15]. These findings show that Nucb2/nesfatin-1 induce apoptosis in ovarian epithelial cell carcinoma through the mTOR/RhoA/ROCK pathway (Figure 1). Surprisingly, this pro-apoptotic effect of Nucb2/nesfatin-1 is contradictory to the effect previously observed for colon and renal cell cancers, where Nucb2/nesfatin-1, through the AMPK/mTOR signaling pathways, contributed to the enhanced aggressiveness and invasiveness of these tumors [19,38]. It is also worth noting that a similar pro-apoptotic effect of Nucb2/nesfatin-1 was reported by Feijóo-Bandín et al. [68] in murine cardiomyocytes. Long stimulation (24 h) of cardiomyocytes with Nucb2/nesfatin-1 brings about apoptosis, which is shown by increased levels of cleaved caspase-3 and decreased levels of phosphorylated Akt—an upstream protein in the mTOR signaling pathway [68].

### 3.2. Nucb2/Nesfatin-1-Induced Cell Death in Adrenocortical Carcinoma

Adrenal cortex tumors (ACTs) are usually benign tumors with an incidence of 3–10% in the population [69]. In contrast, adrenocortical cell carcinomas (ACCs) are a rare type of cancer with a poor prognosis due to their late diagnosis and resistance to chemotherapy [70]. This stems from the fact that ACCs exhibit a downregulated apoptosis pathway with underexpression of genes such as those encoding executioner caspases-3, -6, and -7, as well as the Bcl2-associated X protein (Bax) [71].

Stimulation of H295R cells with Nucb2/nesfatin-1 for 24h induced apoptosis in this cell line in a concentration-dependent manner [72]. Interestingly, Nucb2/nesfatin-1 was shown to induce overexpression of pro-apoptotic genes, such as Bax, in H295R cells (Figure 3). This overexpression was accompanied by a simultaneous decrease in the expression of mRNAs of anti-apoptotic proteins, such as Bcl-2 and Bcl-xL (see Figure 1), but with no significant changes to the expression pattern of the p53 gene or to cellular Ca^2+^ levels [72]. Furthermore, Nucb2/nesfatin-1 alter the phosphorylation pattern of kinases belonging to the family of serine/threonine mitogen-activated protein kinases (MAPKs), resulting in elevated levels of phosphorylated (p-) c-Jun N-terminal kinases 1 and 2 (p-JNK-1/2), and p38^MAPK^ (p-p38^MAPK^), and decreased levels of phosphorylated extracellular signal-regulated kinases 1 and 2 (p-ERK1/2) [72]. MAPKs are involved in the cellular response to extracellular stimuli and their transduction to intracellular effectors. This family of protein kinases is engaged in the control of proliferation, differentiation, apoptosis, and cellular responses to stress [73]. Ras is an upstream protein in the ERK/MAPK pathways, and belongs to the family of small GTPases. A gain-of-function mutations in the *ras* proto-oncogene, which result in an indefinitely active Ras protein along with upregulation of the ERK/MAPK pathways, is often a hallmark of carcinogenesis [73,74]. On the other hand, the JNK-1/2 and p38^MAPK^ pathways are involved in the control of a cell’s response to stress stimuli, and also the regulation of immune responses [75]. Additionally, the JNK-1/2 pathway plays an important role in stress-induced apoptosis [75]. The above findings suggest that Nucb2/nesfatin-1, through upregulation of the JNK-1/2/p38^MAPK^ pathways and downregulation of the Ras/Raf/MEK/ERK pathways, induces apoptosis (Figure 1) in H295R cells in a Ca^2+^-independent manner. Ramanjaneya et al. [72] also reported localization of Nucb2/nesfatin-1 in human adrenocortical cells. Interestingly, Nucb1 and Nucb2 can be proteolytically cleaved by caspase-3, -6, and -8, in vitro and in vivo (Figure 1B) [48]. This fact further underlines the importance of Nucb2 in the control of apoptosis. The putative cleavage site is localized in the region of the first EF-hand motif between residues 235–255 [48]. The effect of the cleavage of Nucb2/nesfatin-1 by caspases on the apoptotic program remains unknown. Nonetheless, Nucb2/nesfatin-1 seems to have an auto-, paracrine and tissue-specific mode of action that could be exploited in the treatment, diagnosis, and monitoring of different types of cancer. Since the Nucb2/nesfatin-1 receptor has not yet been discovered, determination of the exact effectors through which this unique polypeptide exerts this vast array of functions is still hindered.

## 4. Nucb2-Protein Interactions in the Regulation of the Tumorigenesis Process

**Figure 4:**
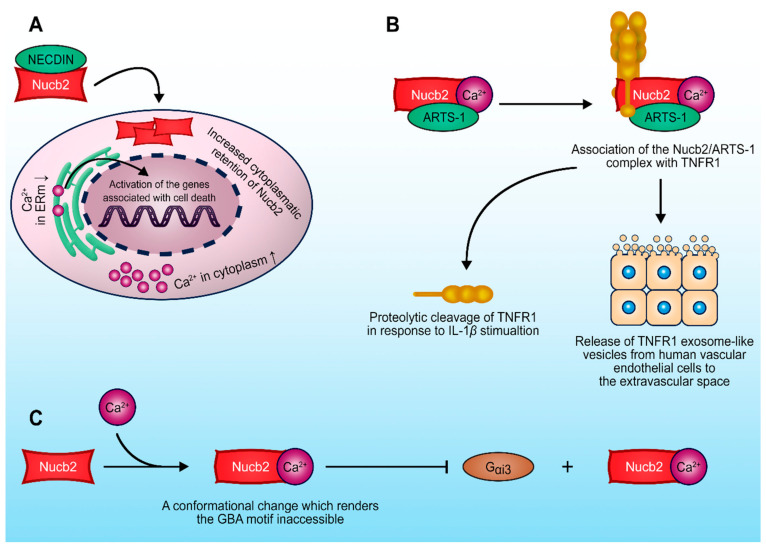
Schematic representation of major Nucb2-protein interactions important in the regulation of the tumorigenesis process. (**A**) Nucb2–necdin interaction disrupts the secretion of Nucb2 and leads to the accumulation of Nucb2 in the cytoplasm. (**B**) Ca^2+^-dependent Nucb2–ARTS-1 interaction leads to the release of both the full-length and truncated forms of TNFR1. (**C**) Suppression of the Nucb2–G_αi3_ interaction occurs due to the Ca^2+^-dependent conformational change of Nucb2’s structure. See the text for more details.

Nucb2 interactions with other proteins deserve special attention [7,8]. Nucb2 has been identified via yeast hybrid assay as a necdin-binding protein (Figure 4A) [8]. The post-mitotic neuron protein necdin is a tumor growth suppressor [76] that interacts with the p53 [77] and E2F1 transcription factors [78]. Taniguchi et al. [8] showed that Nucb2 binds to necdin through the regions of two EF-hand domains and the acidic region [8]. The expression of the necdin protein in cells disrupted the secretion of Nucb2, and led to the accumulation of Nucb2 in the cytoplasm [8]. Remarkably, the Nucb2–necdin interaction increased the level of Ca^2+^ in the cytoplasm [8]. Nucb2 is a Ca^2+^-binding protein, which probably plays a Ca^2+^-sensor protein role [9]. Additionally, Nucb2 is localized in the Golgi apparatus [51], which is a storage compartment of Ca^2+^ [79]. Thus, it appears that Nucb2–necdin interaction, probably through regulation of the Ca^2+^ level, influences Ca^2+^-dependent biological processes, e.g., apoptosis [8]. 

The tumor necrosis factor (TNF) and tumor necrosis factor receptors (TNFRs) belong to the superfamilies of trimeric cytokines and receptors, respectively [80]. TNFRs that contain characteristic cysteine-rich domains include TNFR1, Fas, nerve growth factor receptor, CD40 and CD27, receptor activator of NF-κB, and many more [81]. TNFRs play a key role in the regulation and responses of the immune system, in proliferation, and also in the execution of the apoptotic program. The aminopeptidase regulator of TNFR1 shedding (ARTS-1) has been shown to take part in the release mechanism of TNFR1. ARTS-1 interacts with TNFR1 through their extracellular domains, and facilitates TNFR-1 release [82]. Interestingly, Nucb2 was shown to interact via its EF domains with ARTS-1 in human vascular endothelial cells through two-yeast hybrid screening and co-immunoprecipitation [7], as described earlier. This interaction (Figure 4B) is Ca^2+^-dependent, and Nucb2 is required for the release of both the full-length and truncated forms of TNFR1 [7]. These findings suggest that Nucb2 participates in the turnover of TNFR1 in cells, as well as indirectly in the modulation of TNF activity, thereby affecting the TNF-induced apoptosis pathway.

A separate group of proteins interacting with Nucb2 are G proteins. It has been presented that Nucb2 and its paralog Nucb1, which possess a GBA motif (short for G-binding and -activating motif), belong to the family of non-receptor proteins with GEF activity [83]. These seven conserved amino acid residues are located at the C-terminal region of the second EF-hand domain in the Nucb2 and Nucb1 sequences [83]. This motif has also been discovered in the Gα-interacting vesicle-associated protein (GIV/Girdin), Daple, and synthetic peptides such as KB-752, which also have GEF activity [83,84,85]. Remarkably, Nucb2 and Nucb1 bind preferentially to the GDP-bound form of G_αi3_ through the GEF motif [83]. Additionally, Nucb1 and Nucb2 enhance the GTPase activity of G_αi3_. Due to the overlapping sites for binding Ca^2+^ and G_αi3_ in the Nucb2 and Nucb1 sequences, the presence of Ca^2+^ through conformational rearrangement suppresses interaction of Nucb2 with G_αi3_ in vitro and in vivo [83]. These data indicate that both Nucb1 and Nucb2 belong to the family of proteins that are implicated in G protein regulation (Figure 4C). The dysfunction and upregulation of both G-protein-coupled receptors (GPCRs) and G proteins play a role in various pathological conditions, e.g., cancer progression and metastasis [86,87,88]. Curiously, the GIV/Girdin proteins were characterized as “rheostats” [85]. “Rheostats” are defined as proteins that enhance the signal transduction initiated by the G protein and GPCRs. Dysregulation of these pathways might be implicated in phenotypic changes that result in cancer metastasis [85,89]. Additionally, it has also been suggested that GIV/Girdin might be utilized as potential biomarkers in various cancers [90,91]. The high expression of GIV/Girdin was positively associated with poorer prognosis in cancer cases [92,93]. Barbazan et al. [94] showed that a high expression of proteins with the GBA motif (i.e., GIV/Girdin, Daple, Nucb1 and Nucb2, etc.) in circulating tumor cells (CTCs) from metastatic colon cancer was correlated with poorer outcomes. Combined expression of GBA motif proteins has an improved prognostic value [94]. It was suggested that other GBA motif proteins—e.g., Nucb2—might also participate in tumorigenesis as “rheostats”, in turn dysregulating the G protein activity [94]. 

## 5. Conclusions and Future Perspectives

The number of studies exploring the participation of Nucb2 in tumorigenesis processes is growing. Despite its role in the control of energy homeostasis, Nucb2 seems to be deeply involved in the widely understood pathogenesis of cancer. Numerous studies have revealed that Nucb2, and its derivative, nesfatin-1, are implicated in cancer progression. The high expression of Nucb2 is associated with key traits of cancer and poor prognoses and outcomes, which suggests that Nucb2 might be utilized as a biomarker and prognostic factor of cancer in the future. It has been shown that Nucb2 is connected, through the regulation of the LKB1/AMPK/TORC1/ZEB1 and Akt/mTOR pathways, to cancer metastasis and various accompanying processes, such as EMT [19,38], ERm stress [42], and CACS [57]. Conversely, Nucb2 and nesfatin-1 have also been presented as apoptosis stimulators in ovarian epithelial carcinoma cells and adrenocortical cancer cells through the mTOR/RhoA/ROCK [15], JNK-1/2/p38^MAPK^, and Ras/Raf/MEK/ERK pathways [72]. These results suggest that the role of Nucb2/nesfatin-1 might be tissue dependent (see Table 1). Interestingly, the mTOR pathway seems to be a downstream pathway for both anti- and pro-apoptotic roles of Nucb2. The complete control mechanisms of these processes have, unfortunately, still not been fully identified. The similar dual nature in cancer development has, however, also been observed for other proteins, e.g., for lysine-specific demethylase 6B (KDM6B) [95]. The oncogenic role of KDM6B in EMT regulation was observed to be mediated via Slug activation [96]. Additionally, high expression of KDM6B was found in prostate cancer cells, and was also correlated with poor prognoses in prostate cancer patients [97]. Both Nucb2 and KDM6B are able to regulate the expression levels of different proteins. Nucb2 is a multidomain protein, containing, among others, a region of basic amino acids, a helix–loop–helix motif, and a leucine zipper. All of these motifs are often present in known transcription factors [1], which suggests that Nucb2 might play the role of a transcription factor in the tumorigenesis process. The interactions of Nucb2 with the necdin protein, TNFR1, and G proteins also seem to confirm its dual role in carcinogenesis. Interestingly, all of these interactions are Ca^2+^-dependent, and associated with the structural rearrangements of the carboxy-terminal part of Nucb2—nesfatin-3. We recently showed that nesfatin-3 possesses IDP properties [9]. The significant flexibility of IDPs facilitates a variety of interactions with molecular partners [98]. Nesfatin-3 might provide the binding surface for other proteins. However, the exact function of nesfatin-3 has not yet been characterized. Thus, Nucb2/nesfatin-1 might be utilized as diagnostic tools, and also used in cancer therapy in the future. The suppression of Nucb2 and/or nesfatin-1 could not only contribute to a patient’s survival, but also improve their quality of life. However, further studies are needed in order to clarify the exact mechanism of Nucb2/nesfatin-1 in tumorigenesis, and to elucidate how to regulate their actions. 

## Author Contributions

A.S. and R.L. wrote the original draft with support from D.B. and A.O., R.L. prepared the figures. All authors reviewed and edited the final version of the manuscript. All authors have read and agreed to the published version of the manuscript.

## Figures and Tables

**Table 1:** Summary of Nucb2/nesfatin-1 involvement in tumorigenesis processes.

Cancer Type	Signaling Pathway	Role of Nucb2	Types of Studies	References
Adrenocortical carcinoma	JNK-1/2/p38^MAPK^ Ras/Raf/MEK/ERK	Apoptosis induction	in vitro	[72]
Bladder cancer cells	MMPs	Cancer migration and invasiveness promotion	in vitro	[26]
Colon cancer	LKB1/AMPK/mTORC1/ZEB1 G protein signaling	EMT promotion and cancer metastasis	in vitro and in vivo	[19,94]
Melanoma cells		Adaptation to ER stress	in vitro and in vivo	[42]
Non-small cell lung carcinoma	FTX/ miR-335-5p/ Akt/mTOR	EMT induction and cancer metastasis	in vitro and in vivo	[39]
Ovarian epithelial cell carcinoma	mTOR/RhoA/ROCK	Apoptosis enhancement	in vitro	[15]
Renal cell carcinoma	AMPK/mTORC1/ZEB1	EMT induction	in vitro and in vivo	[38]

## Abbreviations

4EBIP1	eukaryotic initiation factor 4E-binding protein
ACC	acetyl-CoA carboxylase
ACCs	adrenocortical cell carcinomas
ACTs	adrenal cortex tumors
AMPK	5’AMP-activated protein kinase
ARTS-1	aminopeptidase regulator of TNFR1 shedding
ATF6	activating transcription factor 6
Bax	Bcl2 associated X protein
CACS	cancer anorexia-cachexia syndrome
ccRCC	clear-cell renal cell carcinoma
CTCs	circulating tumor cells
EMT	epithelial–mesenchymal transition
ER	estrogen receptor
ERm	endoplasmic reticulum
ERE	estrogen response element
GAPs	GTPase-activating proteins
GDIs	guanine nucleotide dissociation inhibitors
GEFs	guanine nucleotide-exchange factors
GIV/Girdin	Gα-interacting vesicle-associated protein
GPCR	G-protein-coupled receptors
IDPs	inherently disordered proteins
KLF4	Krüppel-like factor 4
LKB1	liver kinase B1
LUAD	lung adenocarcinoma
MAPKs	serine/threonine mitogen activated protein kinases
MMPs	matrix metalloproteinase
mTOR	mammalian target of rapamycin
mTORC1	mTOR complex 1
Nucb2	Nucleobindin-2
ERK1/2	extracellular signal-regulated kinases 1 and 2
JNK-1/2	c-Jun N-terminal kinases-1 and -2
PSA	prostate specific antigen
PVN	paraventricular nucleus
RhoA	Ras homologue gene family member A
ROCKs	Rho-associated coiled-coil-containing kinases
Slug	snail family zinc finger 2
S6K	S6 kinase
S1P	site-1 protease
TNF	tumor necrosis factor
TNFRs	tumor necrosis factor receptors
TSC2	tuberos sclerosis complex 2
Twist	twist family bHLH transcription factor 1
UPR	unfolded protein response
ZEB1	zinc finger E-box-binding homeobox transcription factor 1
